# Current and Future Point-of-Care Tests for Emerging and New Respiratory Viruses and Future Perspectives

**DOI:** 10.3389/fcimb.2020.00181

**Published:** 2020-04-29

**Authors:** Philipp P. Nelson, Barbara A. Rath, Paraskevi C. Fragkou, Emmanouil Antalis, Sotirios Tsiodras, Chrysanthi Skevaki

**Affiliations:** ^1^Institute of Laboratory Medicine, Universities of Giessen and Marburg Lung Center (UGMLC), Philipps University Marburg, German Center for Lung Research (DZL) Marburg, Marburg, Germany; ^2^Vienna Vaccine Safety Initiative - Pediatric Infectious Diseases and Vaccines, Berlin, Germany; ^3^UMR Chrono-Environnement, Université Bourgogne Franche-Comté, Besançon, France; ^4^ESCMID Study Group for Respiratory Viruses (ESGREV), Basel, Switzerland; ^5^4th Department of Internal Medicine, Attikon University Hospital, National and Kapodistrian University of Athens, Athens, Greece

**Keywords:** virus diagnostics, innovative approaches, biosensors, bedside testing, POCT, commercial point-of-care tests

## Abstract

The availability of pathogen-specific treatment options for respiratory tract infections (RTIs) increased the need for rapid diagnostic tests. Besides, retrospective studies, improved lab-based detection methods and the intensified search for new viruses since the beginning of the twenty-first century led to the discovery of several novel respiratory viruses. Among them are human bocavirus (HBoV), human coronaviruses (HCoV-HKU1, -NL63), human metapneumovirus (HMPV), rhinovirus type C (RV-C), and human polyomaviruses (KIPyV, WUPyV). Additionally, new viruses like SARS coronavirus (SARS-CoV), MERS coronavirus (MERS-CoV), novel strains of influenza virus A and B, and (most recently) SARS coronavirus 2 (SARS-CoV-2) have emerged. Although clinical presentation may be similar among different viruses, associated symptoms may range from a mild cold to a severe respiratory illness, and thus require a fast and reliable diagnosis. The increasing number of commercially available rapid point-of-care tests (POCTs) for respiratory viruses illustrates both the need for this kind of tests but also the problem, i.e., that the majority of such assays has significant limitations. In this review, we summarize recently published characteristics of POCTs and discuss their implications for the treatment of RTIs. The second key aspect of this work is a description of new and innovative diagnostic techniques, ranging from biosensors to novel portable and current lab-based nucleic acid amplification methods with the potential future use in point-of-care settings. While prototypes for some methods already exist, other ideas are still experimental, but all of them give an outlook of what can be expected as the next generation of POCTs.

## Introduction

Respiratory viruses such as influenza A viruses (IAV) or human respiratory syncytial virus (RSV) are well-known, circulate worldwide, and are associated with significant morbidity and mortality (Iuliano et al., 2018; Shi et al., 2019). On the other hand, there are emerging infectious diseases which were, according to the definition by the WHO, hitherto unknown or rare but are now rapidly spreading either in number of cases or geographically (WHO, 2014a). In the last 20 years, in addition to the emergence of novel influenza and coronaviruses, advances in molecular detection methods have led to the discovery of new respiratory viruses already circulating worldwide (Jartti et al., 2012).

In 2001 human metapneumovirus (HMPV) was discovered (van den Hoogen et al., 2001). The outbreaks of severe acute respiratory syndrome coronavirus (SARS-CoV) in 2003, Middle East respiratory syndrome coronavirus (MERS-CoV) since April 2012, and SARS-CoV-2 since December 2019 highlight the danger of emerging (zoonotic) and highly pathogenic respiratory viruses (Fouchier et al., 2003; Zaki et al., 2012; WHO, 2020c). Two other coronaviruses, human coronaviruses (HCoV) NL63 and HKU1 were discovered in 2004 and 2005, respectively (Fouchier et al., 2004; van der Hoek et al., 2004; Woo et al., 2005). In 2005, Allander et al. described a new member of the family *Parvovirida*e, human bocavirus (HBoV) (Allander et al., 2005). The two new human polyomaviruses KI and WU (KIPyV, WUPyV) were first discovered in 2007 (Allander et al., 2007; Gaynor et al., 2007) and although often detected in samples, their role in causality of respiratory illness is still under discussion (reviewed in Jartti et al., 2012). In 2007, the human rhinovirus group C (RV-C) was introduced when newly sequenced strains differed significantly from the existing groups A and B (Lee et al., 2007). Finally, avian influenza viruses (AIV) such as IAV H5N1, H7N7, or H9N2 crossed the species barrier to infect humans several times in the last years (reviewed in Kim et al., 2016).

Lab-based techniques still dominate the field of virus diagnostics. Classical methods such as virus cultures, electron microscopy, and serology have been complemented by nucleic acid amplification tests (NAATs), sequencing (including next generation sequencing), and different antigen detection methods. Today, in most clinical settings, NAATs have replaced virus cultures as the gold standard, due to their high specificity, faster turnaround times, and absence of limitations posed by the need for susceptible cell lines (Fox, 2007).

In contrast to lab-based tests, point-of-care tests (POCTs) are performed at the site of sample collection (e.g., bedside, physician's office, or emergency department) and provide results usually in <2 h (Basile et al., 2018; Vos et al., 2019). Furthermore, they require only little hands-on time and no specific laboratory training as most critical steps are automated in a single device. The latter may range from handheld to benchtop size and is not designed for high-throughput sample processing. POCTs and other fast diagnostic tests performed in laboratories but provide results within 1–2 h may be called near-POCTs. Prompt identification of the causative pathogen may help the responsible healthcare professional choosing the appropriate treatment or take the right decisions in outbreak situations, regarding hospitalization and quarantine (Brendish et al., 2015).

In this review, we provide a brief overview of currently available POCTs for the diagnosis of emerging and new respiratory viruses along with their advantages and limitations and discuss recently published approaches and techniques with a potential use in future POCTs.

## Commercially Available Test Systems

For the diagnosis of commonly encountered respiratory viruses such as IAV, influenza B virus (IBV), and RSV many commercially available POCTs and near-POCTs with different sensitivities and specificities for each virus are available (Supplementary Table 1). However, a recent meta-analysis has demonstrated that three newer generation rapid multiplex polymerase chain reaction systems (mPCRs) (*bioMérieux*BioFire® FilmArray® RP, Nanosphere Verigene® RV+ test, and Hologic Gen-Probe Prodesse assays) are highly accurate, though usually more expensive, and may provide important diagnostic information for early identification of IAV, IBV, and RSV (Huang H. S. et al., 2018; Rabold and Waggoner, 2019).

Diagnosis of emerging and novel viruses, including HBoV, RV-C, coronaviruses (e.g., HCoV-HKU1, HCoV-NL63, SARS-CoV-2) as well as specific subtypes of AIV (H5N1, H7N9, H10N8) and reassortant IAV strains remains challenging (Schuster and Williams, 2018; WHO, 2020a). The diagnosis of most of these viruses is based on molecular techniques that can only be performed at specialized referral centers. Recently, there has been increased interest in using POCTs in other settings, such as emergency departments, although implementation might be hampered by the need for specific training (Bouzid et al., 2020). NAATs have higher sensitivity than immunochromatographic assays, but generally, they require a higher degree of technical skills and training (Drancourt et al., 2016). Polymerase chain reaction (PCR) remains the gold standard technique for the diagnosis of AIV subtypes while WHO recommends against the utilization of rapid tests in avian flu diagnosis (WHO, 2005; Monne et al., 2008; Schuster and Williams, 2018). RV-C, is typically detected from nasopharyngeal specimens using reverse transcriptase PCR (RT-PCR) and specific species and serotypes can be further identified by semi-nested PCRs or sequencing (Bochkov et al., 2011; Jartti et al., 2012; Schuster and Williams, 2018).

Diagnosis of HBoV was based on the detection of the specific IgM antibody along with a 4-fold increase of the IgG titer or low IgG avidity indicative of seroconversion, but adaptive immune response requires several hours or even days to develop, thus the utility of such tests in acute settings is limited (Soderlund-Venermo et al., 2009). As HBoV DNA persists in airway secretions for months after an acute infection, quantitative PCR along with serology are currently the preferred diagnostic methods (Christensen et al., 2019). Viral DNAemia, mRNA detection via RT-PCR, and antigen immunodetection assays have shown some promising results but further studies to define their sensitivity, specificity, and applicability in clinical practice are needed (Soderlund-Venermo et al., 2009; Christensen et al., 2010, 2013, 2019; Proenca-Modena et al., 2011; Xu et al., 2017).

The detection of KIPyV and WUPyV, found in secretion from both symptomatic and asymptomatic patients, is based on PCR and serology testing (Neske et al., 2010; Touze et al., 2010; Jartti et al., 2012).

SARS-CoV-2, HCoV-NL63, and -HKU1 as well as HMPV diagnosis is based primarily on RT-PCR methods (van den Hoogen et al., 2004; Nichols et al., 2008; Jartti et al., 2012; WHO, 2020a). No data is available for fluorescent antibody, rapid cultures and enzyme immunoassay (EIA) diagnostic tests for coronaviruses NL63 and HKU1, whereas fluorescent antibody assays may be of some utility for HMPV (Nichols et al., 2008; Jartti et al., 2012). For HMPV specifically, multiplex ligation-dependent probe amplification (MLPA) on a nasopharyngeal swab has high sensitivity and specificity rates (100 and 96%, respectively) (Reijans et al., 2008; Panda et al., 2014; Hoppe et al., 2016).

Available tests for the now extinct SARS-CoV include antibody testing using an EIA and RT-PCR tests in respiratory, blood, and stool specimens (CDC, 2004). For the detection of MERS-CoV, that has its epicenter in the Arabian peninsula, the United States Centers for Disease Control and Prevention (CDC) and the WHO recommend sampling from the lower respiratory tract and real-time RT-PCR (rRT-PCR) testing with specific primers since this appears to be more sensitive than testing of upper respiratory tract specimens (WHO, 2014b,c; CDC, 2015).

NAATs have been proven to be highly accurate and easily scalable tests during large outbreaks of novel or emerging viruses, like the SARS-CoV-2 pandemic. Rapid genome sequencing analysis accommodates the fast development of reliable in-house and commercially available NAATs reagents shortly after an outbreak onset (CDC, 2020; WHO, 2020b).

## Innovative Approaches for Future POCTs

### Biosensors

Biosensors can be a reliable and cost-effective way to detect specific pathogens in point-of-care settings. Different types of sensors for rapid identification of respiratory viruses have been developed recently. By using a gold-coated array of carbon electrodes, the authors were able to detect MERS-CoV spike protein in the picogram range within 20 min (Layqah and Eissa, 2019). This electrochemical assay is based on the competitive binding of a MERS-CoV antibody either to the virus in the sample or to the immobilized antigen on the electrode, which can be measured by a reduced peak current through the chip. In theory, this technique can be easily expanded to simultaneously detect multiple viruses, however, its diagnostic performance needs to be validated with the use of patient samples.

Different types of biosensors for AIV detection use nanobio hybrid materials (reviewed in Lee et al., 2018). In one of those approaches, a DNA probe coupled to a field-effect transistor enabled detection of target DNA down to 1 fM (Lin et al., 2009). Another sensitive technique is based on surface plasmon resonance (SPR), in which biomolecules bound to a metal surface lead to the reduction of the reflection of an incident light beam (Tang et al., 2010; Chang et al., 2018). With a new antibody against a recombinant AIV H7N9, the authors were able to reach a detection limit of a few hundred copies per mL nasal fluid within 10 min of processing time. Although still experimental, the characteristics of this approach render it a promising candidate for a future rapid POCT.

### New Techniques and Prototypes

In a capillary convective PCR (CCPCR), the reagents circulate across a temperature gradient in a simple capillary tube, which allows run times shorter than 30 min (Chou et al., 2011). Together with a self-made dipstick detection method, this principle was already used to test for non-respiratory viruses like hepatitis C virus (Zhang et al., 2013, 2014). Zhou et al. integrated this method into a 1.5 kg device for the automation of the detection of different IAV strains (Zhuo et al., 2018). Although fast and sensitive, manual RNA extraction limits the use as POCT so far. Hardick et al. presented another portable NAAT device not only for the detection of IAV, but also for IBV, RSV, and MERS-CoV. It is based on a RT-PCR in microfluidic cards but likewise lacks automated nucleic acid extraction (Hardick et al., 2018).

Alternatively to nucleic acid-based techniques, giant magnetoresistive (GMR) biosensors function comparable to an enzyme-linked immunosorbent assay but use magnetic labels instead of enzymes or fluorophores coupled to detection antibodies (Hall et al., 2010). With such a sensor, Wu et al. constructed a handheld device which, in connection with a computer or smartphone, was able to detect IAV H3N2 in purified and disrupted virus solutions (Wu et al., 2017). Although H3N2 strains are circulating already since 1968 in the human population (Smith et al., 2004), this method is likely adaptable to emerging AIVs with the use of appropriate capture antibodies.

By designing a prototype for a lateral flow assay for approximately 5 US$, Huang et al. proved that the simultaneous detection of IAV and IBV in swab samples is possible at very low costs (Huang et al., 2017). However, the sensitivity of this prototype needs significant improvements before it can be used under clinical conditions. Instead of constructing a completely new apparatus, Cui et al. used commercially available glucose test strips together with specifically designed glucose-bearing substrates to test for the cleavage activity of IAV neuraminidase in spiked samples (Cui et al., 2017).

Although the majority of the presented approaches and prototypes focuses on the detection of influenza viruses, most of them can theoretically be applied to emerging or new viruses with only minor changes.

### Lab-Based NAATs With Potential Use as Point-of-Care Applications

In comparison to PCRs, isothermal NAATs do not require complex devices when working with extracted nucleic acids. Reverse transcription strand invasion-based amplification (RT-SIBA) and reverse transcription loop-mediated isothermal amplification (RT-LAMP) are two examples which have been used to detect e.g., HMPV (Song et al., 2014), IAV (Eboigbodin et al., 2016), and MERS-CoV (Huang P. et al., 2018). Wang et al. went on to integrate seven RT-LAMP assays into a microfluidic chip for the multiplex detection of different respiratory viruses in a device weighing <3 kg (Wang et al., 2018). Another chip-based system, named iROAD, uses reverse transcription-based recombinase polymerase amplification (RT-RPA) and was able to rapidly identify IAV, different HCoVs, and other respiratory viruses in extracted nucleic acids (Koo et al., 2017).

## Discussion

### Clinical Performance of POCTs

The development of new laboratory and point-of-care diagnostic tests for influenza, RSV, and emerging respiratory viruses has taken up pace in recent years. Healthcare professionals, hospital managers, and laboratory directors will need to update and re-evaluate best practices regularly.

The advancement of diagnostic capabilities may change the way we identify, document, and communicate respiratory viral infections in the future. Along with these developments, the expectations of patients and healthcare professionals may change as well, i.e., patients will want to know the responsible pathogen and their clinical prognosis. With the development of specific antiviral therapies and vaccines, new diagnostic algorithms will be needed to ensure the highest quality of care while containing costs.

From a viewpoint of quality of care and clinical management, timely infection control, and the ability to act upon results, the future will likely belong to portable, CLIA-waived rapid diagnostic tests that take 10–20 min.

A key criterion for the evaluation of diagnostic tests will be their clinical utility, i.e., their ability to identify the current culprit for the patient's symptoms, and to distinguish relevant pathogen(s) from bystander pathogens. More research is needed to correlate clinical outcomes and laboratory data.

A second concern will be the correct timing of diagnostic testing with regards to a patient's course of illness. The sensitivity and positive predictive value of a diagnostic test depend on specimen quality and virus load (which is usually higher in children and early in the course of illness), duration of viral shedding, and patient's immune status. Future diagnostic algorithms will need to consider these factors in addition to the epidemiology of viruses in the respective season or region.

### Advantages and Limitations of POCTs

The initial criticism of POCTs was directed toward a lack of sensitivity and a high degree of variability in test results. Early studies during the 2009/10 flu pandemic reported sensitivities for influenza POCTs ranging from 20 to 70% for the same test kit (Rath et al., 2012). In published evaluation studies, it was not entirely clear whether POCTs were performed at the bedside or whether samples were sent to the laboratory, which would constitute a near-POCT. These methodological inconsistencies also impaired the value of meta-analyses comparing the point-of-care performance of different commercial tests (Chartrand et al., 2015). The reported performance differences also hint to the fact that the training and experience of the staff performing the test have an impact. Procedural concerns were most pronounced with early-stage lateral flow (“strip”) tests, where the result was read manually. Second-generation antigen POCTs and modern molecular POCTs achieve significantly higher reproducibility and sensitivity through automation of key steps in the process. In 2018, the FDA implemented new performance regulations setting new sensitivity thresholds required for POCTs to maintain approval (FDA, 2017). As a result, only several influenza POCTs were taken off the market. The role of regulators in setting quality standards for POCTs cannot be underestimated (Zhang et al., 2016; Azar and Landry, 2018).

In addition to the reliability of the POCT result itself, the hands-on time and the time-to-result are still critical for user-acceptance at the bedside. It is expected that future POCTs will be more robust and easier to use.

As the majority of acute respiratory infections (ARI) is of viral origin, these infections are common reasons for inappropriate antibiotic use (Harris et al., 2016; Tief et al., 2016). Studies have raised the expectation that expanded use of virus diagnostics at the point-of-care may help to limit the use of antibiotics (Bonner et al., 2003). The European Health Action Plan on AMR (European Parliament, 2018) and the O'Neill Report on “Tackling Drug-resistant infections globally” (O'Neill, 2016) both point to rapid diagnostics as a key instrument in tackling AMR. Including POCTs in antimicrobial stewardship programs might increase their acceptance by physicians.

The ability to direct “the right treatment to the right patient at the right time” facilitates precision medicine. In the future, advanced virus diagnostics may be combined with biomarker POCTs for the prediction of individual-level host responses to further target treatment to those who are most likely to benefit from it.

A major obstacle to expanding the use of POCTs is cost. In fact, POCTs would be most impactful in settings where the majority of early treatment decisions are made. The current pricing schemes, however, seem too high for broad-scale testing in the community and emergency departments. In addition to pricing, reimbursement strategies need to be reconsidered. Even in hospital emergency rooms, per-capita standard reimbursements disincentivize the use of virus diagnostics in patients with ARI. Up-to-date economic models are needed to clarify the cost-effectiveness of different types of POCTs.

The steepest increase in antimicrobial resistance is being observed in low-middle income countries (LMIC) (WHO-PAHO, 2018). For LMIC, portable POCTs with multi-modality for known and emerging pathogens, or simple lab-based instrumentation with no/minimal need for cold chain or refrigeration of reagents, may be the most likely to succeed.

### Priorities for the Development of New POCTs

None of the currently commercially available POCTs covers all viruses discussed here (Tables 1, 2, Supplementary Table 1). To date, the same holds true for new technologies as well. However, it is conceivable that these techniques may be adapted to other respiratory viruses after having shown their usefulness in practice. Especially biosensors have the potential for a wider spectrum of applications. They also do not encounter the major limitation of NAATs as POCTs: the extraction of nucleic acids (Ali et al., 2017). For these tests, even less obvious complications, like the stability of the plastic materials against required chemicals, have to be overcome for future highly specific nucleic acid-based POCTs.

**Table 1:** Selected commercial nucleic acid-based point-of-care tests (ordered by time to result).

**POCT commercial name**	**Method/Time to result (min)**	**Detection of new and emerging respiratory viruses: Sensitivity (%)/Specificity(%)**									
		**HBoV**	**SARS-CoV**	**SARS-CoV-2**	**MERS-CoV**	**HCoV-HKU1**	**HCoV-NL63**	**KIPyV, WUPyV**	**HMPV**	**RV-C**	**Emerging IAV**
Cepheid Xpert® Xpress Flu/RSV^b^^,^^c^^1^	rRT-PCR/20-30	N/A	N/A	N/A	N/A	N/A	N/A	N/A	N/A	N/A	H1N1pdm09, H3N2, AIV (H5N2, H5N8) 97.8/100
Mesabiotech™ Accula™ Influenza A&B and RSV^a^^2^	RT-PCR/30	N/A	N/A	N/A	N/A	N/A	N/A	N/A	N/A	N/A	H1N1pdm09, H3N2 97/94
Sekisui Diagnostics Silaris™ Influenza A&B Test^3^	PCR/30	N/A	N/A	N/A	N/A	N/A	N/A	N/A	N/A	N/A	H1N1pdm09, H3N2 97/94
Cepheid Xpert® Flu/RSV XC^b^^,^^c^	rRT-PCR/40-63	N/A	N/A	N/A	N/A	N/A	N/A	N/A	N/A	N/A	H1N1pdm09, H3N2, AIV (H5N2, H5N8) •
(Cepheid Xpert® Xpress SARS-CoV-2, 2020)^e^	rRT-PCR/ 45	N/A	N/A	•	N/A	N/A	N/A	N/A	N/A	N/A	N/A
BioMérieuxBioFire® FilmArray® Respiratory Panel 2 *plus*^4^	Endpoint melt curve analysis/45	N/A	N/A	N/A	•	95.8/99.8	95.8/100	N/A	94.6/99.2	N/A	H1N1pdm09, H3N2 88.9-100/99.6-100
Quidel® Solana® Respiratory Viral Panel Influenza A&B and RSV & HMPV^a^^5^	RT-HDA/45	N/A	N/A	N/A	N/A	N/A	N/A	N/A	•	N/A	*Strains not specified* •
DiaSorin Simplexa™ Flu A/B & RSV Direct Kit^6^	RT-PCR/60	N/A	N/A	N/A	N/A	N/A	N/A	N/A	N/A	N/A	H1N1pdm09, H3N2, >20 AIVs, 2 swine influenza strains 97/97.9
Cepheid Xpert® Flu^b^	rRT-PCR/75	N/A	N/A	N/A	N/A	N/A	N/A	N/A	N/A	N/A	H1N1pdm09 97.1-100/99.6
Luminex Verigene® RP Flex Test^7^	RT-PCR/ <120	N/A	N/A	N/A	N/A	N/A	N/A	N/A	•	N/A	H1, H3 •
GenMark Dx® ePlex® Respiratory Panel^d^	PCR/ <120	N/A	N/A	N/A	N/A	•	•	N/A	N/A	N/A	H1N1pdm09, H3N2 •
(GenMark Dx ePlex® SARS-CoV-2 Test, 2020) Test^e^	rRT-PCR/ <120	N/A	N/A	•	N/A	N/A	N/A	N/A	N/A	N/A	N/A
(QIAGEN, 2019) Panel v2	rRT-PCR/120	100/99.5	N/A	N/A	N/A	100/100	91.7/100	N/A	100/100	N/A	H1N1pdm09, H3N2 91.7-100/100

1 *Cepheid Xpert Flu. Product Page. Available online at: https://www.cepheid.com/en/cepheid-solutions/clinical-ivd-tests/critical-infectious-diseases/xpert-flu (accessed September 13, 2019)*.

2 *Mesabiotech™ Accula™ Flu A/FluB Test Package Insert. Manual. Available online at: https://static1.squarespace.com/static/5ca44a0a7eb88c46af449a53/t/5cddacb3f9689300010d93c3/1558031549318/LBL-60010+Accula+Flu+A+Flu+B+Package+Insert+EU.pdf (accessed November 10, 2019)*.

3 *Sekisui Diagnostics Silaris™ Influenza A&B Test. Manual. 481. Available online at: https://sekisuidiagnostics.com/product-documents/60012-d_v1.5_silaris_ifu.pdf (accessed November 10, 2019)*.

4 *Biomerieux BIOFIRE^®^ FILMARRAY^®^ Panels. Product Page. Available online at: https://www.biomerieux-diagnostics.com/filmarrayr-respiratory-panel (accessed September 13, 2019)*.

5 *Quidel^®^ Solana Respiratory Viral Panel. Product Page. Available online at: https://www.quidel.com/molecular-diagnostics/respiratory-viral-panel (accessed November 10, 2019)*.

6 *DiaSorin Simplexa^®^ Flu A/B and RSV Direct Kit. Product Page. Available online at: https://molecular.diasorin.com/us/kit/simplexa-flu-ab-rsv-direct-kit/ (accessed November 10, 2019)*.

7 *Luminex Verigene® Respiratory Pathogens Flex Test. Product Page. Available online at: https://www.luminexcorp.com/respiratory-pathogens-flex-test/ (accessed September 13, 2019)*.

*N/A, Virus not included in this assay; • Virus included, but specificity/sensitivity not available; RT-HAD, Reverse transcriptase helicase-dependent amplification; RT-PCR, Reverse transcriptase polymerase chain reaction; rRT-PCR, real-time RT-PCR; HBoV, Human bocavirus; (H)CoV, (Human) coronavirus; KI/WUPyV, KI/WU polyomavirus; HMPV, Human metapneumovirus; RV-C, Human rhinovirus type C; IAV, Influenza A virus; IBV, Influenza B virus; H1N1pdm09, Influenza A H1N1 pandemic 2009; AIV, Avian influenza virus; RSV, Respiratory Syncytial virus*.

*Please refer to Supplementary Table 1 for detailed reliability parameters*.

a *Two different kits for Influenza (A, B) and RSV*;

b *Performance reviewed in Basile et al. (2018)*;

c *Described in Loeffelholz et al. (2019)*;

d *Performance tested by Babady et al. (2018)*;

e *For use under the Emergency Use Authorization (EUA) only*.

**Table 2:** Selected commercial antigen-based point-of-care tests (ordered by time to result).

**POCT commercial name**	**Method/Time to result (min)**	**Detection of new and emerging respiratory viruses: Sensitivity (%)/Specificity(%)**									
		**HBoV**	**SARS-CoV**	**SARS-CoV-2**	**MERS-CoV**	**HCoV-HKU1**	**HCoV-NL63**	**KIPyV, WUPyV**	**HMPV**	**RV-C**	**Emerging IAV**
BD Veritor™ System Influenza A+B and RSV^a^^,^^b^^8^	LFIC/Digital immunoassay/10-11	N/A	N/A	N/A	N/A	N/A	N/A	N/A	N/A	N/A	H1N1pdm09, H3N2, AIV (H5N1, H5N2, N7N9) 89.6-90.2/99.07
Abbott SD BIOLINE Influenza Ag A/B/A(H1N1) pandemic^b^^9^	CI/10-15	N/A	N/A	N/A	N/A	N/A	N/A	N/A	N/A	N/A	H1N1pdm09 54.5-91.8/96.8-100
Princeton BioMeditech BioSign® Rapid Flu A+B Antigen Panel Test^10^	ICMI/10-15	N/A	N/A	N/A	N/A	N/A	N/A	N/A	N/A	N/A	H1N1pdm09, H3N2v, AIV (H5N1, H7N9) 89.2/99.4
Quidel® QuickVue® Influenza A+B and RSV^a^^,^^b^^11^	LFIC/10-15	N/A	N/A	N/A	N/A	N/A	N/A	N/A	N/A	N/A	H3N2, variable performance against other strains 20-98/89-100
Abbott Alere BinaxNOW® Influenza A+B and RSV^a^^,^^b^^12^	LFIC/15	N/A	N/A	N/A	N/A	N/A	N/A	N/A	N/A	N/A	H3 44.8-83/93-100
Quidel® Sofia® Influenza A+B Fluorescent Immunoassay^13^	FIA/15	N/A	N/A	N/A	N/A	N/A	N/A	N/A	N/A	N/A	H1N1pdm09, H3N2, variable performance against other strains 90-99/95-96
Thermo Scientific™ Xpect™ Influenza A+B and RSV^a^^14^	LFIC/15	N/A	N/A	N/A	N/A	N/A	N/A	N/A	N/A	N/A	H1N1pdm09, H3N2, AIV (H5N1, H7N9, H9N2) 92.2/100
ArcDia mariPOC® Respi^b^^15^	PE/20-120	76.5/100	N/A	N/A	N/A	N/A	N/A	N/A	78/100	N/A	H1N1v, H2N2, H3N2, AIV (H5N1, H7N9, H9N2, H7N3) 92.3-100/99.8-100

8 *BDVeritor™ Flu A+B. Product Page. Available online at: https://www.bd.com/en-us/offerings/capabilities/microbiology-solutions/point-of-care-testing/veritor-system (accessed September 13, 2019)*.

9 *Abbot SD BIOLINE Influenza Ag A/ B/ A(H1N1) Pandemic. Product Page. Available online at: https://www.alere.com/en/home/product-details/sd-bioline-influenza-ag-aba-pandemic.html (accessed September 13, 2019)*.

10 *Princeton BioMeditech BioSign® Flu A+B. Product Page. Available online at: http://pbm.pequod.com/pages/products/biosign_flu_ab (accessed November 10, 2019)*.

11 *Quidel® QuickVue® Influenza A+B Test. Manual. Available online at: https://www.quidel.com/sites/default/files/product/documents/EF1350313EN00_1.pdf (accessed September 13, 2019)*.

12 *Abbot Alere Binax NOW®. Product Page. Available online at: https://www.alere.com/en/home/products-services/brands/binaxnow.html (accessed September 13, 2019)*.

13 *Quidel® Sofia® Influenza A+B FIA. Manual. Available online at: https://www.quidel.com/sites/default/files/product/documents/EF1219109EN00.pdf (accessed September 13, 2019)*.

14 *Thermo Scientific™ Xpect™ Flu A and B, and RSV. Brochure. Available online at: https://assets.thermofisher.com/TFS-Assets/MBD/brochures/Xpect-Flu-RSV-Brochure-991-135-ENG.pdf (accessed November 10, 2019)*.

15 *ArcDia mariPOC® respi. Brochure. Available online at: https://www.arcdia.com/wp-content/uploads/2019/05/2019-03-mariPOC-Respi-brochure-EN.pdf (accessed September 13, 2019)*.

*N/A, Virus not included in this assay; FIA, Fluorescent immunoassay; LFIC, Lateral flow immunochromatography assay; CI, Chromatographic immunoassay; PE, Photofluorescent excitation; ICMI, Immunochromatographic membrane immunoassay; HBoV, Human bocavirus; (H)CoV, (Human) coronavirus; KI/WUPyV, KI/WU polyomavirus; HMPV, Human metapneumovirus; RV-C, Human rhinovirus type C; IAV, Influenza A virus; H1N1pdm09, Influenza A H1N1 pandemic 2009; AIV, Avian influenza virus; IBV, Influenza B virus; RSV, Respiratory Syncytial virus*.

*Please refer to Supplementary Table 1 for detailed reliability parameters*.

a *Two different kits for Influenza (A, B) and RSV*;

b *Performance reviewed in Basile et al. (2018)*.

Any ideal POCT should fulfill the ASSURED criteria of the World Health Organization to be applicable in resource-limited settings (Mabey et al., 2004). Currently, this is most likely true for tests based on lateral flow immunochromatography but coming improvements e.g., in miniaturization and battery capacity may facilitate the use of other test principles (Basile et al., 2018).

While economic benefits of POCTs and better outcomes for the patients are still discussed, it is likely that these tests will gain further importance with decreasing processing costs and improved robustness.

## Author Contributions

CS and PN planned, structured, and edited the manuscript. PN searched the literature and integrated all contributions. PN, BR, PF, EA, and ST participated in the writing of the first draft of the manuscript and subsequent revisions. BR and PF contributed equally to this work. All authors critically read, reviewed, and approved the submitted final version of the manuscript.

## Conflict of Interest

CS received consultancy fees and research funding from Hycor Biomedical and Thermo Fisher Scientific, research funding from Mead Johnson Nutrition (MJN), and consultancy fees from Bencard Allergie. The remaining authors declare that the research was conducted in the absence of any commercial or financial relationships that could be construed as a potential conflict of interest.
