# Supplementary material for: Current and Future Point-of-Care Tests for Emerging and New Respiratory Viruses and Future Perspectives
Source: Front Cell Infect Microbiol. 2020 Apr 29;10:181. doi: 10.3389/fcimb.2020.00181 (PMC7202255; doi:10.3389/fcimb.2020.00181)
Supplement: Supplementary file 1 [file Table_1.docx]

Supplementary Material

**Supplementary Table 1.** Characteristics of selected point-of-care tests (POCTs).

| **POCT COMMERCIAL NAME** | **METHOD** | **TIME TO RESULT (min)** | **VIRUSES TESTED** | **SENSITIVITY (%)** | **SPECIFICITY (%)** | **PPA (%)** | **NPA (%)** | |
| --- | --- | --- | --- | --- | --- | --- | --- | --- |
| **NUCLEIC ACID-BASED POCTs** | | | | | | | | |
| **Abbott Alere™ i (ID Now™) Influenza A&B and RSV***  (*Abbot Alere ID NOW™;* Basile et al., 2018) | LAMP NEAR | 13-15 | Influenza A, B  RSV (A, B) | Flu A (77.8-97.9)  Flu B (75-92)  RSV (98.6-100) | Flu A (62.5-100)  Flu B (53.6-99)  RSV (97-98) | N/A | N/A | |
| **Roche Cobas® Liat® Influenza A/B & RSV** (*Roche cobas® Liat® Influenza A/B & RSV;* Basile et al., 2018) | rRT-PCR | 20 | Influenza A, B  RSV | N/A | N/A | Flu A (98.4-99.6)  Flu B (97.9-99.35)  RSV (96.8-97.8) | Flu A (96.5)  Flu B (99.4)  RSV (98.4) | |
| **Roche Cobas® Influenza A/B** *(Roche cobas® Influenza A/B Assay, 2019)* | rRT-PCR | 20 | Influenza A,B | Flu A (97.5)  Flu B (96.9) | Flu A (97.9)  Flu B (97.9) | N/A | N/A | |
| **Cepheid Xpert® Xpress Flu/RSV** *(Basile et al., 2018; Loeffelholz et al., 2019)* | rRT-PCR | 20-30 | Influenza A, B  RSV (A, B) | Flu A (97.8)  Flu B (100)  RSV (90.6) | Flu A (100)  Flu B (99.4)  RSV (99.4) | Flu A (100)  Flu B (100)  RSV (97.1) | Flu A (95.2)  Flu B (99.5)  RSV (99.6) | |
| **Cepheid Xpert® Xpress SARS-CoV-2** *(Cepheid Xpert® Xpress SARS-CoV-2, 2020)* **^#^** | rRT-PCR | 45 | SARS-CoV-2 | N/A | N/A | SARS-CoV-2 (100) | SARS-CoV-2 (100) | |
| **Genesystem GENECHECKER™ Ultra-Fast PCR System and Rapi:chip™ (Chip-based ultra-fast PCR with own primers)** *(Kwon et al., 2018)* | RT-PCR | 23 | Influenza A, B | N/A | N/A | N/A | N/A | |
| **Mesabiotech™ Accula™ Influenza A&B and RSV*** (*Mesabiotech™ Accula™ Flu A/Flu B Test Package Insert; Mesabiotech™ Accula™ RSV Test*) | RT-PCR | 30 | Influenza A (H1N1, H3N2), Influenza B  RSV | Flu A (97)  Flu B (94)  RSV (90.2) | Flu A (94)  Flu B (99)  RSV (95.6) | N/A | N/A | |
| **Sekisui Diagnostics Silaris^TM^ Influenza A&B Test** (*Sekisui Diagnostics Silaris™ Influenza A&B Test*) | PCR | 30 | Influenza A (including H1N1pdm09, H3N2), Influenza B | Flu A (97)  Flu B (94) | Flu A (94)  Flu B (99) | N/A | N/A | |
| **Sekisui Diagnostics Silaris^TM^ RSV Test** *(Sekisui Diagnostics Silaris™ RSV Test)* | PCR | 30 | RSV | 90.2% | 95.6% | N/A | N/A | |
| **Cepheid Xpert® Flu/RSV XC** (Basile et al., 2018; Loeffelholz et al., 2019) | rRT-PCR | 40-63 | Influenza A, B  RSV (A, B) | N/A | N/A | Flu A (85.7-100)  Flu B (92-100)  RSV (98.5 -100) | Flu A (98.9-100)  Flu B (100)  RSV (99.6 -100) | |
| **BioMérieux BioFire® FilmArray® Respiratory Panel 2 *plus*** *(Biomerieux BIOFIRE® FILMARRAY® RP Panel)* | Endpoint melt curve analysis | 45 | AdV  HCoV (229E, HKU1, OC43, NL63)  MERS-CoV  Influenza A (including H1N1pdm09,H1N1, H3N2), Influenza B  HMPV  PIV 1-4  RSV  RV/EV | Flu A (88.9-100)  Flu B (100)  RSV (100)  AdV (88.9-100)  RV/EV (92.7-95.7)  PIV 1 (97.1-100)  PIV2 (87.4-100)  PIV 3 (95.8-100)  PIV 4 (100)  HCoV-HKU1 (95.8)  HCoV-NL63 (95.8)  HCoV-229E (100)  HCoV-OC43 (100) | Flu A (99.6-100)  Flu B (100)  RSV (89.1)  AdV (99.8)  RV/EV (94.6)  PIV 1(99.9)  PIV2 (99.8)  PIV 3(99.8)  PIV 4 (99.9)  HCoV-HKU1 (99.8)  HCoV-NL63 (100)  HCoV-229E (99.8)  HCoV-OC43 (99.6) | N/A | N/A | |
| **Quidel® Solana® Respiratory Viral Panel Influenza A&B and RSV & HMPV*** (*Quidel® Solana® Respiratory Viral Panel*) | RT-HDA | 45 | Influenza A, B  RSV  HMPV | N/A | N/A | Flu A (97.2)  Flu B (100)  RSV (95.5)  HMPV (95.6) | Flu A (96.7)  Flu B (98.9)  RSV (99.9)  HMPV (99.8) | |
| **Quidel® Lyra® SARS-CoV-2 Assay** *(Quidel® Lyra® SARS-CoV-2 Assay)*^†^ | rRT-PCR | 75 (expected) | SARS-CoV-2 | N/A | N/A | SARS-CoV-2 (100) | SARS-CoV-2 (100) | |
| **Janssen Diagnostics/Biocartis Idylla™ Respiratory (IFV-RSV)**  (*Janssen Diagnostics/Biocartis Idylla^™^ IFV - RSV*) | rRT-PCR | 50 | Influenza A (including H1N1pdm09, H1N1, H3N2, H275Y oseltamivir mutation), Influenza B, RSV(A, B) | N/A | N/A | N/A | N/A | |
| **BioMérieux BioFire® FilmArray® Respiratory Panel** (*Biomerieux BIOFIRE® FILMARRAY® RP Panel;* Basile et al., 2018) | Endpoint melt curve analysis | 60 | AdV  HBoV  CoV (229E, HKU1, OC43, NL63, MERS)  Influenza A (including H1N1pdm09,H1N1, H3N2), Influenza B  HMPV  PIV 1-4  RSV  RV1-4 (not C)/EV | Flu A (90-100)  Flu B (100)  RSV (100)  AdV (88.9-100)  HBoV (66.7-100)  RhV/EV (92.7-92.9)  PIV 1 (100)  PIV2 (87.4-90)  PIV 3 (95.8-100)  PIV 4 (100) | Flu A (99.1-99.8)  Flu B (100)  RSV (89.1-100)  AdV (99.2-100)  HBoV (98.4-99.8)  RhV/EV (94.6-96.2)  PIV 1(99.2-99.9)  PIV2 (99.8-100)  PIV 3(99.8-100)  PIV 4 (99.9-100) | N/A | N/A | |
| **BioMérieux BioFire® FilmArray® Respiratory Panel 2** (Leber et al., 2018) | Endpoint melt curve analysis | 60 | AdV  HCoV (229E, HKU1, OC43, NL63)  Influenza A (including H1N1pdm09,H1N1, H3N2), Influenza B  HMPV  PIV 1-4  RSV  RV/EV | N/A | N/A | Flu A (100)  Flu B (100)  RSV (99.40)  AdV (94.6)  RV/EV (97.5)  PIV 1 (100)  PIV2 (97.9)  PIV 3 (95.6)  PIV 4 (100)  HCoV-229E (91.7)  HCoV-HKU1 (100)  HCoV-OC43 (80.5)  HCoV-NL63 (100)  HMPV (97.3) | Flu A (100)  Flu B (99.9)  RSV (98.3)  AdV (96.9)  RV/EV (93.5)  PIV 1 (99.9)  PIV 2 (99.5)  PIV 3 (99.4)  PIV 4 (99.6)  HCoV-229E (99.7)  HCoV-HKU1 (99.2)  HCoV-OC43 (99.7)  HCoV-NL63 (99.4)  HMPV (99.5) | |
| **DiaSorin Simplexa™ Flu A/B & RSV Direct Kit** (*DiaSorin Simplexa® Flu A/B & RSV Direct Kit*) | RT-PCR | 60 | Influenza A (including H1, H1N1pdm09, H3), Influenza B  RSV | Flu A (97)  Flu B (100)  RSV (97.6) | Flu A (97.9)  Flu B (99.9)  RSV (92.9) | N/A | N/A | |
| **DiaSorin Simplexa™ COVID-19 Direct** (*DiaSorin Simplexa™ COVID-19 Direct*)**^#^** | rRT-PCR | N/A | SARS-CoV-2 | N/A | N/A | SARS-CoV-2 (100) | SARS-CoV-2 (100) | |
|  |  |  |  |  |  |  |  | |
|  |  |  |  |  |  |  |  | |
|  |  |  |  |  |  |  |  | |
|  |  |  |  |  |  |  |  | |
|  |  |  |  |  |  |  |  | |
|  |  |  |  |  |  |  |  | |
| **Cepheid Xpert® Flu** (*Cepheid Xpert® Flu;* Basile et al., 2018) | rRT-PCR | 75 | Influenza A, B | Flu A (90-100)  Flu B (100)  A/H1N1pdm09 (97.1-100) | Flu A (95.2-98.1)  Flu B (99.1-99.6)  A/H1N1pdm09 (99.6) | N/A | N/A | |
| **Enigma Diagnostics Enigma® MiniLab Respiratory Viral Panel (RVP) Test** *(Enigma® MiniLab Respiratory Viral Panel (RVP) Test, 2019)* | RT-PCR | 90 | Influenza A, B  RSV A, B | N/A | N/A | Flu A (84.8)  Flu B (92)  RSV (99.4) | Flu A (100)  Flu B (100)  RSV (100) | |
| **Luminex ARIES® Flu A/B & RSV** (*Luminex ARIES® Flu A/B & RSV Assay Overview*) | rRT-PCR | <120 | Influenza A, B  RSV | N/A | N/A | Flu A (95.8)  Flu B (93.8)  RSV (97.1) | Flu A (98.4)  Flu B (99.4)  RSV (98.4) | |
| **Luminex Verigene® RP Flex Test** (*Luminex Verigene® Respiratory Pathogens Flex Test.*) | RT-PCR | <120 | AdV  Influenza A (including H1, H3), Influenza B  HMPV  PIV 1-4  RSV (A, B)  RhV |  |  | Flu A (98.3)  A/H1 (97.8)  A/H3 (100)  Flu B (98)  RSV A (100)  RSV B (100)  AdV (86)  PIV 1 (90.0)  PIV2 (92.3)  PIV 3 (82.4)  PIV 4 (79.2)  HMPV (100)  RhV (81.9) | Flu A (99.4)  A/H1 (99.7)  A/H3 (99.8)  Flu B (99.6)  RSV A (99.9)  RSV B (98.8)  AdV (97.2)  PIV 1 (99.9)  PIV2 (99.9)  PIV 3 (99.9)  PIV 4 (99.8)  HMPV (99.7)  RhV (97.1) | |
| **QIAGEN QIAstat-Dx® Respiratory Panel v2** *(QIAGEN, 2019)* | rRT-PCR | 120 | AdV  HBoV  HCoV (229E, HKU1, OC43, NL63)  Influenza A (including H1N1pdm09, A/H1, A/H3), Influenza B  HMPV (A, B)  PIV 1-4  RSV (A, B)  RV/EV | Flu A (91.7-100)  Flu B (98.2)  RSV (97.1)  AdV (97.2)  HBoV (100)  RV/EV (94.9)  PIV 1 (100)  PIV2 (100)  PIV 3 (100)  PIV 4 (83.3)  HCoV-229E (80)  HCoV-HKU1 (100)  HCoV-OC43 (100)  HCoV-NL63 (91.7)  HMPV (100) | Flu A (100)  Flu B (100)  RSV (100)  AdV (99.5)  HBoV (99.9)  RV/EV (98.4)  PIV 1(100)  PIV2 (100)  PIV 3(100)  PIV 4 (99.9)  HCoV-229E (100)  HCoV-HKU1 (100)  HCoV-OC43 (100)  HCoV-NL63 (100)  HMPV (100) | N/A | N/A | |
| **Hologic® Panther Fusion® Flu A/B/RSV** *(Hologic® Panther Fusion® Flu A/B/RSV, 2019)*^†^ | rPCR | 145 | Influenza A (including H1N1pdm09, H3N2), Influenza B  RSV | Flu A (98.8)  Flu B (100)  RSV (99.3) | Flu A (99)  Flu B (99.8)  RSV (99) | Flu A (98.8)  Flu B (100)  RSV (99.3) | Flu A (99)  Flu B (99.8)  RSV (99) | |
| **Hologic® Panther Fusion® AdV/HMPV/RV** *(Hologic® Panther Fusion® AdV/hMPV/RV, 2019)*^†^ | rPCR | 145 | AdV  HMPV  RV | AdV (97.9)  HMPV (98.7) | AdV (97.6)  HMPV (99) | AdV (97.9)  HMPV (98.7) | AdV (97.6)  HMPV (99) | |
| **Hologic® Panther Fusion® Paraflu** *(Hologic® Panther Fusion® Paraflu, 2019)*^†^ | rPCR | 145 | PIV 1-4 | PIV 1 (97.1)  PIV2 (91.7)  PIV 3 (96.3)  PIV 4 (96.7) | PIV 1 (99.6)  PIV2 (99.5)  PIV 3 (99)  PIV 4 (99.8) | PIV 1 (97.1)  PIV2 (91.7)  PIV 3 (96.3)  PIV 4 (96.7) | PIV 1 (99.6)  PIV2 (99.5)  PIV 3 (99)  PIV 4 (99.8) | |
| **Hologic® Panther Fusion® SARS-CoV-2** (*Hologic® Panther Fusion® SARS-CoV-2 Assay, 2020*)**^#,^**^†^ | rRT-PCR | 145 (expected) | SARS-CoV-2 | N/A | N/A | SARS-CoV-2 (100) | SARS-CoV-2 (100) | |
| **LG Life Sciences AdvanSure™** *(Jung et al., 2015)*^†^ | rPCT | 180 | AdV  HBoV  HCoV (229E, OC43, NL63)  Influenza A, Influenza B  HMPV  PIV 1-3  RSV (A,B)  RV | AdV (100)  FluA (100),  Flu B (100)  RSV B (100)  RV (73.8) | AdV (100)  FluA (100),  Flu B (100)  RSV B (95.1)  RV (88.9) | AdV (100)  HCoV-229E/NL63 (60)  FluA (100), Flu B (100)  HMPV (100)  PIV 1(100)  PIV 2 (50)  PIV 3 (100)  RSV A(100)  RSV B (100)  RV (60.3) | AdV (91.1)  HCoV-229E/NL63 (91.8)  FluA (98.9), Flu B (99.8)  HMPV (100)  PIV 1(99.8)  PIV 2 (100)  PIV 3 (99.8)  RSV A(99.6)  RSV B (98.5)  RV (95.9) | |
| **Luminex® NxTAG® Respiratory Pathogen Panel** *(Chan et al., 2017; Luminex NxTAG® Respiratory Pathogen Panel, 2019)* | RT-PCR | 240 | AdV  HBoV  HCoV (229E, HKU1, OC43, NL63)  Influenza A (including H1, H3),  Influenza B  HMPV  PIV 1-4  RSV (A,B)  RV/EV | Flu A (100)  Flu B (100)  RSV A (100)  RSV B (100)  AdV (100)  HBoV (100)  RV/EV (92.9)  PIV 1 (100)  PIV2 (90)  PIV 3 (100)  PIV 4 (72.7)  HCoV-229E (100)  HCoV-HKU1 (100)  HCoV-OC43 (100)  HCoV-NL63 (100)  HMPV (90.2) | Flu A (99.1)  Flu B (100)  RSV A (99.2)  RSV B (100)  AdV (99.2)  HBoV (98.4)  RV/EV (96.2)  PIV 1 (99.2)  PIV2 (100)  PIV 3 (100)  PIV 4 (100)  HCoV-229E (100)  HCoV-HKU1 (100)  HCoV-OC43 (100)  HCoV-NL63 (100)  HMPV (100) | N/A | N/A | |
| **GenMark Dx® eSensor® Respiratory Viral Panel** *(Pierce and Hodinka, 2012; GenMark Dx Respiratory Viral Panel, 2019)* | eSensor® Technology | 360 | AdV (B/E,C)  Influenza A (including H1N1pdm09, H1N1, H3N2), Influenza B  HMPV  PIV 1-3  RSV (A, B)  RV/EV | AdV (100)  Flu A (100)  Flu B (100)  HMPV (100)  RSV A (100)  RSV B (100)  PIV 1 (100)  PIV 2 (100)  PIV 3 (100)  RV/EV (97.5) | N/A | AdV (90.3)  Flu A (97.2)  Flu B (91.7)  HMPV (92)  RSV A (100)  RSV B (95.8)  PIV 1 (95.8)  PIV 2 (100)  PIV 3 (91.7)  RV/EV (97.1) | AdV (100)  Flu A (100)  Flu B (100)  HMPV (100))  RSV A (100)  RSV B (100)  PIV 1 (100)  PIV 2 (100)  PIV 3 (100)  RV/EV (95.8) | |
| **Abbott Molecular PLEX-ID Flu Assay** *(Tang et al., 2013)*^†^ | RT-PCR/EMI-MS | N/A | Influenza A (including H1, H1N1pdm09, H3), Influenza B | N/A | N/A | Flu A (94.5)  Flu B (96)  H1N1pdm09 (98.3) | Flu A (99)  Flu B (99.9)  H1N1pdm09 (97.5) | |
| **GenMark Dx® ePlex® Respiratory Panel** (*GenMark Dx Respiratory Pathogen (RP) Panel;* Babady et al., 2018) | PCR | <120 | AdV  HCoV (229E, HKU1, OC43, NL63)  Influenza A (including H1N1pdm09, H1N1, H3N2), Influenza B  HMPV  PIV 1-4  RSV (A, B)  RV/EV | N/A | N/A | Flu A (93.8)  A/H1 (-)  A/H1pdm09 (95.1)  A/H3 (89.8)  Flu B (89.6)  RSV A (89.6)  RSV B (94.1)  AdV (93.2)  HCoV (85.1)  PIV 1 (91.8)  PIV2 (91.8)  PIV 3 (91)  PIV 4 (92.9)  HMPV (93.3)  RV/EV (94.6) | Flu A (99.9)  A/H1 (100)  A/H1pdm09 (99.8)  A/H3 (100)  Flu B (99.8)  RSV A(100)  RSV B (99.9)  AdV (98.9)  HCoV (99.5)  PIV 1 (99.9)  PIV2 (99.9)  PIV 3 (99.8)  PIV 4 (99.8)  HMPV (99.8)  RV/EV (95.7) | |
| **GenMark Dx® ePlex® SARS-CoV-2 Test (***GenMark Dx ePlex®SARS-CoV-2 Test***)^#^** | rRT-PCR (eSensor® technology) | <120 | SARS-CoV-2 | N/A | N/A | SARS-CoV-2 (94.4) | SARS-CoV-2 (100) | |
| **Nanosphere Verigene® RV+ Test** (*Nanosphere Verigene® Respiratory Virus Plus Nucleic Acid Test on the Verigene® System*) | RT-PCR | N/A | Influenza A (including H1, H1N1pdm09, H3), Influenza B  RSV (A, B) | Flu A (98.7)  A/H1 (100)  A/H1pdm09 (99.5)  A/H3 (100)  Flu B (100)  RSV (97.2) | Flu A (93.2)  A/H1 (99.9)  A/H1pdm09 (100)  A/H3 (100)  Flu B (99.7)  RSV (99.5) | N/A | N/A | |
| **NON NUCLEIC ACID-BASED POCTs** | | | | | | | | |
| **BD Veritor™ System Influenza A+B and RSV*** (*BD Veritor™ Flu A+B;* Basile et al., 2018) | LFIC / Digital Immunoassay | 10-11 | Influenza A, H1N1pdm09, H3N2, H5N1, H5N2, H7N9), Influenza B  RSV | Flu A (89.6-90.2)  Flu B (87.5-98.8)  RSV (61.3 -91.6) | Flu A (99.07)  Flu B (100)  RSV (94.5-100) | N/A | N/A | |
| **Abbott SD BIOLINE Influenza Ag A/B/A(H1N1) pandemic**  (*Abbot SD BIOLINE Influenza Ag A/ B/ A(H1N1) Pandemic;* Basile et al., 2018) | CI | 10-15 | Influenza A (including H1N1pdm09), Influenza B | Flu A & B (54.5-91.8) | Flu A & B (96.8-100) | N/A | N/A | |
| **Princeton BioMeditech BioSign® Rapid Flu A+B Antigen Panel Test** (*Princeton BioMeditech BioSign® Flu A+B*) | ICMI | 10-15 | Influenza A, B | Flu A (89.2)  Flu B (86.4) | Flu A (99.4)  Flu B (99) | N/A | N/A | |
| **Quidel QuickVue® Influenza A+B and RSV*** (*Quidel® QuickVue® Influenza A+B Test;* Basile et al., 2018) | LFIC | 10-15 | Influenza A (including H1N1pdm09, H3N2), Influenza B  RSV | Flu A (20-98)  Flu B (21-87)  RSV (58-92) | Flu A (89-100)  Flu B (90-100)  RSV(90-100) | N/A | N/A | |
| **Abbott Alere BinaxNOW® Influenza A+B and RSV*** (*Abbot Alere BinaxNOW®;* Basile et al., 2018) | LFIC | 15 | Influenza A (including A/H3 and A/H1), Influenza B  RSV | Flu A (44.4-83)  Flu B (25-70.8)  RSV (41-93) | Flu A (93-100)  Flu B (96-100)  RSV (82-100) | N/A | N/A | |
| **BD Directigen™ EZ RSV and Flu A + B*** (*BD Directigen^™^ EZ Flu A+B; BD Directigen™ EZ RSV;* Basile et al., 2018) | LFIC | 15 | Influenza A, B  RSV | Flu A,B (20.6-62.2)  RSV (66.7-87.2) | Flu A,B (97.1-100)  RSV (85.5-95) | N/A | N/A | |
| **Fujirebio ESPLINE® Influenza A&B-N**  (Basile et al., 2018) | EIA | 15 | Influenza A, B | Flu A (86.8-91.2)  Flu B (88.2-94.4) | Flu A (95.8-97.1)  Flu B (98-99.7) | N/A | N/A | |
| **Quidel® Sofia® influenza A+B fluorescent immunoassay** *(Quidel® Sofia® Influenza A+B FIA; Rath et al., 2012; Tuttle et al., 2015)* | FIA | 15 | Influenza A (including H1N1pdm09, H3N2), Influenza B | Flu A (90-99)  Flu B (88-90) | Flu A (95-96)  Flu B (96-97) | N/A | N/A | |
| **Thermo Scientific™ Xpect™ Influenza A+B and RSV*** (*Thermo Scientific™ Xpect™ Flu A&B and RSV*)] | LFIC | 15 | Influenza A, B  RSV | Flu A (92.2)  Flu B (97.8)  RSV (95.6) | Flu A (100)  Flu B (100)  RSV (94.1) | N/A | N/A | |
| **ArcDia mariPOC® Quick Flu/RSV** (*ArcDia mariPOC® Quick Flu/RSV*) | PE | 20 | Influenza A, B  RSV | N/A | N/A | N/A | N/A | |
| **ArcDia mariPOC® Respi** (*ArcDia mariPOC® respi;* Basile et al., 2018) | PE | 20-120 | Influenza A (including H1N1v, H1N1, H3N2, H5N1, H7N9, H2N2, H9N2, H7N3),  Influenza B  RSV (A, B)  HMPV (A, B)  PIV 1,2,3  HCoV-OC43  AdV  HBoV | Flu A (92.3-100)  Flu B (88-100)  RSV (89-100)  HMPV (78)  HBoV (76.5)  HCoV-OC43  AdV (100) | Flu A (99.8-100)  Flu B (100)  RSV (100)  HMPV (100)  HBoV (100)  H-CoV-OC43  AdV (100) | N/A | N/A | |
| N/A: Data not available, CI: Chromatographic Immunoassay, FIA: Fluorescent Immunoassay, LFIC: Lateral Flow Immunochromatography Assay, LAMP: Loop mediated isothermal amplification, ICMI: Immunochromatographic Membrane Immunoassay, NEAR: Nicking Enzyme Amplification Reaction, PE: Photofluorescent Excitation, RT-HDA: Reverse Transcriptase-Helicase Dependent Amplification, RT-PCR: Reverse Transcriptase Polymerase Chain Reaction, rRT-PCR: real-time Reverse Transcriptase Polymerase Chain Reaction, qPCR: quantitative Polymerase Chain Reaction, rPCR: real-time PCR, EIA: Enzyme Immunoassay, EMI-MS: Electrospray Ionization Mass Spectrometry, Flu A: Influenza A, Flu B: Influenza B, RSV: Respiratory syncytial virus, HMPV: Human metapneumovirus, PIV: Parainfluenza virus, AdV: Adenovirus, HCoV: Human coronavirus, RV/EV: Rhino/Enteroviruses, HBoV: Human bocavirus, PPA: Positive Percent Agreement, NPA: Negative Percent Agreement, H1N1pdm09: Influenza A H1N1 pandemic 2009.  *** Two different kits for Influenza A, B and RSV. ^#^ For use under the Emergency Use Authorization (EUA) only,** ^†^ Rapid PCR kit or test for high-throughput platform.  ***Note****: None of these commercial POCTs detect the following emerging viruses: SARS-CoV, , KIPyV and WUPyV.* | | | | | | | |  |

REFERENCES

*Abbot Alere BinaxNOW®*. Product page. Accessed September 13, 2019, <https://www.alere.com/en/home/products-services/brands/binaxnow.html>

*Abbot Alere ID NOW™*. Product page. Accessed September 13, 2019, <https://www.alere.com/en/home/product-details/id-now.html>

*Abbot SD BIOLINE Influenza Ag A/ B/ A(H1N1) Pandemic*. Product page. Accessed September 13, 2019, <https://www.alere.com/en/home/product-details/sd-bioline-influenza-ag-aba-pandemic.html>

*ArcDia mariPOC® Quick Flu/RSV*. Brochure. Accessed September 13, 2019, <https://www.arcdia.com/wp-content/uploads/2019/05/2019-03-mariPOC-Quick-brochure-EN.pdf>

*ArcDia mariPOC® respi*. Brochure. Accessed September 13, 2019, <https://www.arcdia.com/wp-content/uploads/2019/05/2019-03-mariPOC-Respi-brochure-EN.pdf>

Babady, N. E., England, M. R., Jurcic Smith, K. L., He, T., Wijetunge, D. S., Tang, Y.-W., et al. (2018). Multicenter Evaluation of the ePlex Respiratory Pathogen Panel for the Detection of Viral and Bacterial Respiratory Tract Pathogens in Nasopharyngeal Swabs. *J Clin Microbiol* 56, e01658-17. doi: 10.1128/JCM.01658-17

Basile, K., Kok, J., and Dwyer, D. E. (2018). Point-of-care diagnostics for respiratory viral infections. *Expert Rev Mol Diagn* 18, 75–83. doi: 10.1080/14737159.2018.1419065

*BD Directigen^™^ EZ Flu A+B*. Brochure. Accessed September 13, 2019, <http://legacy.bd.com/resource.aspx?IDX=12124>

*BD Directigen™ EZ RSV*. Brochure. Accessed September 13, 2019, <http://legacy.bd.com/resource.aspx?IDX=12123>

*BD Veritor™ Flu A+B*. Product page. Accessed September 13, 2019, <https://www.bd.com/en-us/offerings/capabilities/microbiology-solutions/point-of-care-testing/veritor-system>

*Biomerieux BIOFIRE® FILMARRAY® RP Panel*. Product page. Accessed September 13, 2019, <https://www.biomerieux-diagnostics.com/filmarrayr-respiratory-panel>

*Cepheid Xpert® Flu*. Product page. Accessed September 13, 2019, <https://www.cepheid.com/en/cepheid-solutions/clinical-ivd-tests/critical-infectious-diseases/xpert-flu>

*Cepheid Xpert® Xpress SARS-CoV-2* (2020). Assay Manual. Accessed March 25, 2020, <https://www.fda.gov/media/136314/download>

Chan, K. H., To, K. K. W., Li, P. T. W., Wong, T. L., Zhang, R., Chik, K. K. H., et al. (2017). Evaluation of NxTAG Respiratory Pathogen Panel and Comparison with xTAG Respiratory Viral Panel Fast v2 and Film Array Respiratory Panel for Detecting Respiratory Pathogens in Nasopharyngeal Aspirates and Swine/Avian-Origin Influenza A Subtypes in Culture Isolates. *Adv Virol* 2017, 1324276. doi: 10.1155/2017/1324276

*DiaSorin Simplexa® Flu A/B & RSV Direct Kit*. Product page. Accessed November 10, 2019, <https://molecular.diasorin.com/us/kit/simplexa-flu-ab-rsv-direct-kit/>

*DiaSorin Simplexa™ COVID-19 Direct*. Assay Manual. Accessed March 25, 2020, <https://www.fda.gov/media/136286/download>

*Enigma® MiniLab Respiratory Viral Panel (RVP) Test* (2019). Manual, <http://www.virotechdiagnostics.com/fileadmin/user_upload/Doc/AL/AL_HWML-CA-0002.pdf>

*GenMark Dx ePlex®SARS-CoV-2 Test (2020).* Assay Manual. Accessed March 25, 2020, https://www.fda.gov/media/136282/download

*GenMark Dx Respiratory Pathogen (RP) Panel*. Product page. Accessed September 13, 2019, <https://www.genmarkdx.com/solutions/panels/eplex-panels/respiratory-pathogen-panel/>

*GenMark Dx Respiratory Viral Panel* (2019). Product page. Accessed November 16, 2019, <https://www.genmarkdx.com/solutions/panels/xt-8-panels/respiratory-viral-panel/>

*Hologic® Panther Fusion® AdV/hMPV/RV* (2019). Manual. Accessed November 16, 2019, <https://www.hologic.com/sites/default/files/2019-11/AW-16834_003_01.pdf>

*Hologic® Panther Fusion® Flu A/B/RSV* (2019). Manual. Accessed November 16, 2019, <https://www.hologic.com/sites/default/files/2019-06/AW-16832_002_01.pdf>

*Hologic® Panther Fusion® Paraflu* (2019). Manual. Accessed November 16, 2019, <https://www.hologic.com/sites/default/files/2019-06/AW-16833_002_01.pdf>

*Hologic® Panther Fusion® SARS-CoV-2 Assay (2020)*. Assay Manual. Accessed March 25, 2020, <https://www.fda.gov/media/136156/download>

*Janssen Diagnostics/Biocartis Idylla^™^ IFV - RSV*. Brochure. Accessed September 13, 2019, <https://media.biocartis.com/biocartis/documents/IFV-RSV_brochure_web.pdf>

Jung, Y. J., Kwon, H. J., Huh, H. J., Ki, C.-S., Lee, N. Y., and Kim, J.-W. (2015). Comparison of the AdvanSure™ real-time RT-PCR and Seeplex(®) RV12 ACE assay for the detection of respiratory viruses. *J Virol Methods* 224, 42–46. doi: 10.1016/j.jviromet.2015.08.003

Kwon, S.-H., Lee, S., Jang, J., Seo, Y., and Lim, H.-Y. (2018). A point-of-care diagnostic system to influenza viruses using chip-based ultra-fast PCR. *J Med Virol* 90, 1019–1026. doi: 10.1002/jmv.25046

Leber, A. L., Everhart, K., Daly, J. A., Hopper, A., Harrington, A., Schreckenberger, P., et al. (2018). Multicenter Evaluation of BioFire FilmArray Respiratory Panel 2 for Detection of Viruses and Bacteria in Nasopharyngeal Swab Samples. *J Clin Microbiol* 56. doi: 10.1128/JCM.01945-17

Loeffelholz, M., Jones, R., Uy, C., Lo, D., Kele, A., and Baron, E. J. (2019). *Cepheid assays detect a broad range of contemporary human and avian influenza strains*. Accessed September 13, 2019, <https://www.cepheid.com/administrator/components/com_productcatalog/library-files/13c07133b4e35667b67e9158de356e05-1353---Monograph-on-Human-Influenza-and-Avian-Strains-Final-08082019-08082021.pdf>

*Luminex ARIES® Flu A/B & RSV Assay Overview*. Product Page. Accessed September 13, 2019, <https://www.luminexcorp.com/aries-flu-ab-rsv-assay/>

*Luminex NxTAG® Respiratory Pathogen Panel* (2019). Product page, <https://www.luminexcorp.com/nxtag-respiratory-pathogen-panel/#overview>

*Luminex Verigene® Respiratory Pathogens Flex Test.* Product page. Accessed September 13, 2019, <https://www.luminexcorp.com/respiratory-pathogens-flex-test/>

*Mesabiotech™ Accula™ Flu A/Flu B Test Package Insert*. Manual. Accessed November 10, 2019, <https://static1.squarespace.com/static/5ca44a0a7eb88c46af449a53/t/5cddacb3f9689300010d93c3/1558031549318/LBL-60010+Accula+Flu+A+Flu+B+Package+Insert+EU.pdf>

*Mesabiotech™ Accula™ RSV Test*. Manual. Accessed November 10, 2019, <https://static1.squarespace.com/static/5ca44a0a7eb88c46af449a53/t/5cddab6c189e750001421ebe/1558031216770/60032-EN+Rev+B+-+Accula+RSV+Package+Insert+EU+EN.pdf>

*Nanosphere Verigene® Respiratory Virus Plus Nucleic Acid Test on the Verigene® System*. Manual. Accessed September 13, 2019, <http://www.nanosphere.us/sites/default/files/support-docs/rv_package_insert.pdf>

Pierce, V. M., and Hodinka, R. L. (2012). Comparison of the GenMark Diagnostics eSensor respiratory viral panel to real-time PCR for detection of respiratory viruses in children. *J Clin Microbiol* 50, 3458–3465. doi: 10.1128/JCM.01384-12

*Princeton BioMeditech BioSign® Flu A+B*. Product page. Accessed November 10, 2019, <http://pbm.pequod.com/pages/products/biosign_flu_ab>

QIAGEN (2019). *QIAstat-Dx® Respiratory Panel Instructions for Use: Version 2*. Manual. Accessed November 10, 2019, <https://www.qiagen.com/ie/resources/resourcedetail?id=371ac902-c220-4e88-9d22-9012c33f268b&lang=en>

*Quidel® Lyra® SARS-CoV-2 Assay* (2020). Assay Manual. Accessed March 26, 2020, https://www.fda.gov/media/136227/download

*Quidel® QuickVue® Influenza A+B Test*. Manual. Accessed September 13, 2019, <https://www.quidel.com/sites/default/files/product/documents/EF1350313EN00_1.pdf>

*Quidel® Sofia® Influenza A+B FIA*. Manual. Accessed September 13, 2019, <https://www.quidel.com/sites/default/files/product/documents/EF1219109EN00.pdf>

*Quidel® Solana® Respiratory Viral Panel*. Product page. Accessed November 10, 2019, <https://www.quidel.com/molecular-diagnostics/respiratory-viral-panel>

Rath, B., Tief, F., Obermeier, P., Tuerk, E., Karsch, K., Muehlhans, S., et al. (2012). Early detection of influenza A and B infection in infants and children using conventional and fluorescence-based rapid testing. *J Clin Virol* 55, 329–333. doi: 10.1016/j.jcv.2012.08.002

*Roche cobas® Influenza A/B Assay*. Product page. Accessed November 16, 2019, <https://diagnostics.roche.com/global/en/products/params/cobas-influenza-a-b-assay.html>

*Roche cobas® Liat® Influenza A/B & RSV*. Product page. Accessed September 13, 2019, <https://www.cobasliat.com/point-of-care-influenza-RSV-test>

*Sekisui Diagnostics Silaris™ Influenza A&B Test*. Manual. Accessed November 10, 2019, <https://sekisuidiagnostics.com/product-documents/60012-d_v1.5_silaris_ifu.pdf>

*Sekisui Diagnostics Silaris™ RSV Test*. Manual. Accessed November 16, 2019, <https://www.sekisuidiagnostics.com/wp-content/uploads/2019/07/Silaris-RSV-Package-Insert.pdf>

Tang, Y.-W., Lowery, K. S., Valsamakis, A., Schaefer, V. C., Chappell, J. D., White-Abell, J., et al. (2013). Clinical accuracy of a PLEX-ID flu device for simultaneous detection and identification of influenza viruses A and B. *J Clin Microbiol* 51, 40–45. doi: 10.1128/JCM.01978-12

*Thermo Scientific™ Xpect™ Flu A&B and RSV*. Brochure. Accessed November 10, 2019, <https://assets.thermofisher.com/TFS-Assets/MBD/brochures/Xpect-Flu-RSV-Brochure-991-135-ENG.pdf>

Tuttle, R., Weick, A., Schwarz, W. S., Chen, X., Obermeier, P., Seeber, L., et al. (2015). Evaluation of novel second-generation RSV and influenza rapid tests at the point of care. *Diagn Microbiol Infect Dis* 81, 171–176. doi: 10.1016/j.diagmicrobio.2014.11.013
